# XMD-17-51 Inhibits DCLK1 Kinase and Prevents Lung Cancer Progression

**DOI:** 10.3389/fphar.2021.603453

**Published:** 2021-03-08

**Authors:** Wei-Qiang Yang, Wei-Jun Zhao, Liu-Lian Zhu, Shuai-Jun Xu, Xue-Lin Zhang, Yong Liang, Xiao-Fei Ding, Alexander Kiselyov, Guang Chen

**Affiliations:** ^1^ Department of Clinical Medicine, School of Medicine, Taizhou University, Taizhou, China; ^2^ Graduate School of Medicine, Hebei North University, Zhangjiakou, China; ^3^Department of Chest, Taizhou Central Hospital, Taizhou University, Taizhou, China; ^4^ Department of Experimental and Clinical Medicine, School of Medicine, Taizhou University, Taizhou, China; ^5^ Department of Pharmaceutical Engineering, School of Pharmaceutical Chemical and Materials Engineering, Taizhou University, Taizhou, China; ^6^ Department of Pharmacology, School of Medicine, Taizhou University, Taizhou, China

**Keywords:** XMD17-51, DCLK1, NSCLC, stem cell, EMT

## Abstract

Doublecortin-like kinase 1 (DCLK1) is a cancer stem cell marker that is highly expressed in various types of human cancer, and a protein kinase target for cancer therapy that is attracting increasing interest. However, no drug candidates targeting DCLK1 kinase have been developed in clinical trials to date. XMD-17-51 was found herein to possess DCLK1 kinase inhibitory activities by cell-free enzymatic assay. In non-small cell lung carcinoma (NSCLC) cells, XMD-17-51 inhibited DCLK1 and cell proliferation, while DCLK1 overexpression impaired the anti-proliferative activity of XMD-17-51 in A549 cell lines. Consequently, XMD-17-51 decreased Snail-1 and zinc-finger-enhancer binding protein 1 protein levels, but increased those of E-cadherin, indicating that XMD-17-51 reduces epithelial-mesenchymal transition (EMT). Furthermore, sphere formation efficiency was significantly decreased upon XMD-17-51 treatment, and XMD-17-51 reduced the expression of stemness markers such as β-catenin, and pluripotency factors such as SOX2, NANOG and OCT4. However, the percentage of ALDH^+^ cells was increased significantly following treatment with XMD-17-51 in A549 cells, possibly due to EMT inhibition. In combination, the present data indicated that XMD-17-51 inhibited DCLK1 kinase activity in a cell-free assay with an IC_50_ of 14.64 nM, and decreased DCLK1 protein levels, cell proliferation, EMT and stemness in NSCLC cell lines. XMD-17-51 has the potential to be a candidate drug for lung cancer therapy.

## Introduction

XMD-17-51 trifluoroacetate, a pyrimido-diazepinone compound, was originally discovered as a derivative of HTH-01-015, which is a selective inhibitor of NUAK1 (Banerjee et al., 2014; Gray et al., 2016). However, XMD-17-51 can inhibit the activity of NUAK1 kinase more effectively, with an IC_50_ of only 1.5 nM. XMD-17-51 has also been shown to inhibit several members of the AMPK family (MARK1, MARK3, BRSK1, and AMPK) and kinases associated with growth and proliferation (Banerjee et al., 2014).

Doublecortin-like kinase 1 (DCLK1) is a serine kinase consisting of a microtubule-binding domain with two double corticosteroid motifs at the N-terminus and a serine/threonine kinase domain at the C-terminus (Patel et al., 2016). In addition, a special serine/proline domain at the N- and C-terminus is essential for regulating the interaction between proteins (Liu et al., 2016). DCLK1, a member of the protein kinase superfamily and the dipcortin family was initially found to be associated with microtubules involved in neurogenesis and neuronal migration (Lin et al., 2000). Recently, DCLK1 has been identified as a novel, tumor-specific stem cell marker in the intestine and pancreas (Nakanishi et al., 2013; Bailey et al., 2014; Chandrakesan et al., 2017). In addition, DCLK1 was found to be highly expressed in several malignancies and to be involved in the regulation of tumorigenesis, tumor stemness and epithelial-mesenchymal transition (EMT) in cancer, including pancreatic, colorectal and liver cancer (Chandrakesan et al., 2014; Weygant et al., 2015; Nguyen et al., 2016; Westphalen et al., 2016; Weygant et al., 2016; Chandrakesan et al., 2017; Liu et al., 2018). However, the expression and biological function of DCLK1 in non-small cell lung carcinoma (NSCLC) remain unclear. Data collected from TCGA database show that the expression of DCLK1 in lung adenocarcinoma tissues is significantly increased, as compared with that in adjacent normal tissues, and is associated with prognosis (Panneerselvam et al., 2020). The potential tumorigenic function of DCLK1 in solid tumors remains largely unknown in NSCLC. Given the close link between DCLK1 and cancer, research on DCLK1 small molecule inhibitors has been progressing in recent years. LRRK2-IN-1 (Weygant et al., 2014), XMD8-92 (Yang et al., 2010) and XMD8-85 (Deng et al., 2011) have been confirmed to have an inhibitory effect on DCLK1. In particular, the discovery of DCLK1-IN-1, a new selective chemical probe for the DCLK1 kinase, has enabled us to comprehensively investigate the precise roles of DCLK1 in cancer (Ferguson et al., 2020). However, DCLK1 kinase inhibitors have neither been evaluated in clinical trials nor been approved for clinical therapy.

In the present study, XMD-17-51 was found to possess DCLK1 kinase inhibitory activities in the cell free enzymatic assay and NSCLC cell lines, including A549, NCI-H1299 and NCI-H1975 cell lines. XMD-17-51 was found to elicit an anti-cancer activity, partly through the inhibition of DCLK1, and be a potential candidate for cancer therapy.

## Materials and Methods

### Reagents

XMD-17-51, polybrene and puromycin were purchased from MCE, 3-(4,5-dimethylthiazol-2-yl)-2,5-diphenyl tetrazolium bromide (MTT; cat. no., M8180) was obtained from Beijing Solarbio Science & Technology Co., Ltd. Antibodies against the mammalian target of DCLK1 (cat. no., 21699-1-AP), extracellular regulated protein kinases (ERK1/2; cat. no., 16443-1-AP), zinc-finger-enhancer binding protein 1 (ZEB1; cat. no., 21544-1-AP), SNAI1 (cat. no., 13099-1-AP), and epithelial cadherin (E-cadherin; cat. no., 20874-1-AP) were obtained from Proteintech Group Inc. Phosphorylated-p44/42 ERK1/2 (p-ERK1/2; cat. no., 4370S) was obtained from Cell Signaling Technology, Inc.

### Cell Culture

A549, NCI-H1299 and NCI-H1975 human lung cancer cell lines and 293T human kidney epithelial cell line were purchased from the Chinese Academy of Sciences Type Culture Collection (CASTCC) and maintained in an appropriate medium, as recommended by the CASTCC. A549 was maintained in RPMI 1640 supplemented with 10% heat-inactivated fetal bovine serum (FBS). NCI-H1299 and NCI-H1975 were cultured in RPMI 1640, supplemented with 10% FBS, 1% sodium pyruvate, and 1x glutamine. 293T was grown in DMEM with 10% FBS. Cells were incubated in a humidified atmosphere of 95% air plus 5% CO_2_ at 37°C.

### *In Vitro* Kinase Assa

Purified kinase-active DCLK1 0.25 μg (cat. no., 02-139, lot. no., 15CBS-0243C; Carna Biosciences, Inc.) was incubated in kinase buffer (50 mM HEPES, pH 7.5; 0.01% Tween-20; 10 mM MgCl_2_; 1 mM EGTA) with 2.5 μg ULight-CREBtide peptide (cat. no., TRF0200-D; lot. no., 2427110; PerkinElmer, Inc.), 1 μM ATP (Merck KGaA), and either DMSO or XMD-17-51 (the compounds were tested from 10 μM, 3-fold dilution, 10 concentration points) for 1 h at 30°C. Subsequently, the detection solution was added at a 1:1 ratio to the reactions, which were gently mixed and then incubated for another 1 h protected from the light. Cells were collected on Envision with excitation at 320 nm and emission at 665 and 615 nm.

### RT-qPCR

Total RNA was extracted with TRIzol reagent, according to the manufacturer’s instructions, and was then reverse-transcribed with Prime Script™ RT reagent Kit (Takara Biotechnology Co., Ltd.). The resultant cDNA was amplified by RT-qPCR using a DCLK1-specific primer pair. The primer sequences were as follows: DCLK1, CAG​CAA​CCA​GGA​ATG​TAT​TGG​A forward and CTC​AAC​TCG​GAA​TCG​GAA​GAC​T, reverse; GAPDH, 5′-GCA​CCG​TCA​AGG​CTG​AGA​AC-3′ forward and 5′-GCC​TTC​TCC​ATG​GTG​GTG​AA-3′ reverse. The thermocycling conditions were as follows: 95°C For 30 s followed by 40 cycles of 95°C for 5 s, 60°C for 30 s, and then 95°C for 15 s, 60°C for 60 s and 95°C for 15 s. Gene expression was assessed by the ΔCt method and mRNA levels of DCLK1 were normalized to the amount of GAPDH mRNA in an identical sample.

### Western Blotting

The cells were collected and lysed. The cell lysates were then analyzed by western blotting. Total protein concentration was determined by BCA protein assay (Thermo Fisher Scientific, Inc.). Protein samples were immunoblotted according to standard procedures. Proteins (40 μg) were separated on 8% SDS-polyacrylamide gels and transferred onto PVDF membranes. The membranes were blocked with 5% skimmed milk powder for 2 h at room temperature, followed by incubation with primary antibodies at 4°C overnight. Subsequently, the membranes were washed with PBS containing 0.1% Tween-20 and then incubated with secondary antibodies for 1 h at room temperature. The blots were visualized using ECL Plus Western Blotting Detection reagents (Beyotime Institute of Biotechnology) and scanned in Image Quant LAS 4000 mini (GE Healthcare Bio-Sciences).

### Small Interfering RNA Transfection

Briefly, 60 pmol DCLK1 siRNA (5′-GGG​AGU​GAG​AAC​AAU​CUA​CTT-3′ forward and 5′-UUC​UCC​GAA​CGU​GUC​ACG​UTT-3′ reverse) or negative control siRNA (Shanghai GenePharma Co., Ltd.) was diluted in 50 μl Opti-MEM I Reduced Serum Medium (Thermo Fisher Scientific, Inc.) without serum (the final concentration of RNA when added to the cells was 100 nM) and mixed gently. In addition, Lipofectamine 2000 (Thermo Fisher Scientific, Inc.) was mixed gently before use, and then 1 μl Lipofectamine 2000 was diluted in 50 μl Opti- MEM I Reduced Serum Medium. It was then mixed gently and incubated for 5 min at room temperature. Next, the diluted oligomer was combined with the diluted Lipofectamine 2000, mixed gently and incubated for 20 min at room temperature. The oligomer-Lipofectamine 2000 complexes were added to 30–50% confluent A549 cells with 500 μl complete medium, followed by gentle mixing by rocking the plate back and forth. The cells were incubated at 37°C in a CO_2_ incubator for 48 h before the gene knockdown assay.

### Lentivirus-Mediated Overexpression

pLenti-DCLK1 and empty vector were obtained from Obio Technology. Cells were seeded in a T25 cell culture flask at a density of 6 × 10^5^ cells/well 24 h before infection. The medium was removed, and a 10–20 multiplicity of infection (MOI) of MISSION pCMV-DCLK1-puro lentiviral particles along with 2 ml of DMEM medium (Thermo Fisher Scientific, Inc.) supplemented with polybrene (8 μg/ml; Sigma-Aldrich) was added to the cells. Lentiviral infection was performed at 37°C for 6 h, unless intensive cell toxicity was observed. The medium was then replaced with 5 ml of fresh medium. Infected cells were allowed to grow for 48–72 h and then selected with puromycin-containing medium (Merck KGaA). The expression of DCLK1 was confirmed by RT-qPCR and western blotting.

### Cell Proliferation

Cell proliferation was assessed by MTT assay. Cells (5 × 10^3^ cells per well) were seeded into a 96-well tissue culture plate in triplicate. The cells were cultured in the presence of XMD-17-51 with DMSO as a vehicle at different concentrations. At 48 h after treatment, 10 μl TACS MTT Reagent (R&D Systems, Inc.) was added to each well and the cells were incubated at 37°C, until dark crystalline precipitate became visible in the cells. A total of 100 µl of 266 mM NH_4_OH in DMSO was added to the wells and placed on a plate shaker at low speed for 5 min. Following shaking, the plate was allowed to incubate for 10 min protected from the light, and the OD550 for each well was read using a microplate reader. The results were averaged and calculated as a percentage of the DMSO (vehicle) control ± the SE of the mean.

### Sphere Formation Assay

For the sphere formation assay, cells (100 cells/well) suspended in stem cell medium, which is DMEM-F12 medium containing 4 U/l insulin, 20 ng/ml EGF, 20 ng/ml basic fibroblast growth factor, and B27 Serum-Free Supplement (50×) were seeded in ultra-low attachment in 96-well plates and incubated at 37°C in a 5% CO_2_ humidified incubator. After 10 days, spheres were counted and photographed under the microscope.

### ALDEFLUOR Assay

ALDEFLUOR assay (STEMCELL Technologies) was performed to distinguish the cancer stem cell (CSC) population in A549 cells, according to the manufacturer’s instructions. In brief, cells were placed in Aldefluor assay buffer containing the ALDH substrate. For the negative control, cells were immediately treated with 1.5 mM diethylamino benzaldehyde, a specific ALDH inhibitor. After 30 min of incubation at 37°C, the population of ALDH^+^ and ALDH^−^ cells were quantified by flow cytometry.

### Statistical Analysis

All statistical analysis was performed using GraphPad Prism version 8.0 (GraphPad Software, Inc.). The results are expressed as the mean ± SD. Student’s t-test and one-way ANOVA was used to identify significant differences between ≥2 groups. *p* < 0.05 was considered to indicate a statically significant difference.

## Results

### XMD-17-51 Inhibits DCLK1 Kinase Activity

**FIGURE 1:**
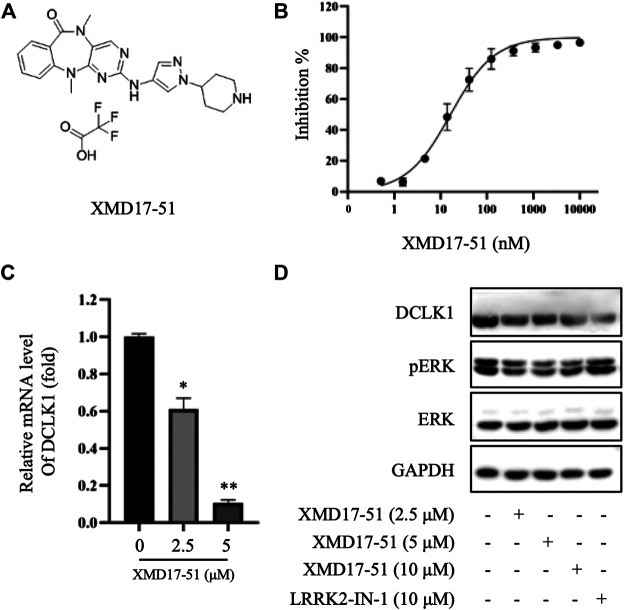
XMD-17-51 inhibits DCLK1 kinase activity. **(A)** Molecular structure of XMD-17-51. **(B)** XMD-17-51 inhibited DCLK1 kinase activity in an *in vitro* kinase assay performed using purified active DCLK1 kinase. **(C)** XMD-17-51 decreased DCLK1 mRNA expression levels in A549 cells, as shown by RT-qPCR (normalization to GAPDH levels). Data are presented as fold change relative to the DCLK1 levels in vehicle control cells. **(D)** DCLK1 and its downstream target protein level in A549 cells were decreased significantly following treatment with XMD-17-51, GAPDH was used as a loading control. The data shown are **(A**,**D)** representative of or **(B**,**C)** are presented as the mean ± SD of at least three independent experiments. DCLK1, doublecortin-like kinase 1.

An *in vitro* cell-free kinase assay was performed using commercially available purified DCLK1 protein and ULight-CREBtide peptide substrate with a low ATP concentration (1 μM). Using this assay, the IC_50_ of XMD-17-51 inhibition of DCLK1 was estimated to be 14.64 nM (Figure 1B). To confirm the inhibition of DCLK1 kinase *in vitro*, A549 cells were treated with XMD-17-51 at different concentrations for 24 h. DCLK1 expression levels were decreased upon XMD-17-51 treatment in a dose-dependent manner (Figures 1C,D). In addition, *p*-ERK (Thr202/Tyr204) was also reduced in both 42 and 44 kDa isoforms with XMD-17-51 treatment (Figure 1D).

### XMD-17-51 Inhibited NSCLC Cell Proliferation Via DCLK1

**FIGURE 2:**
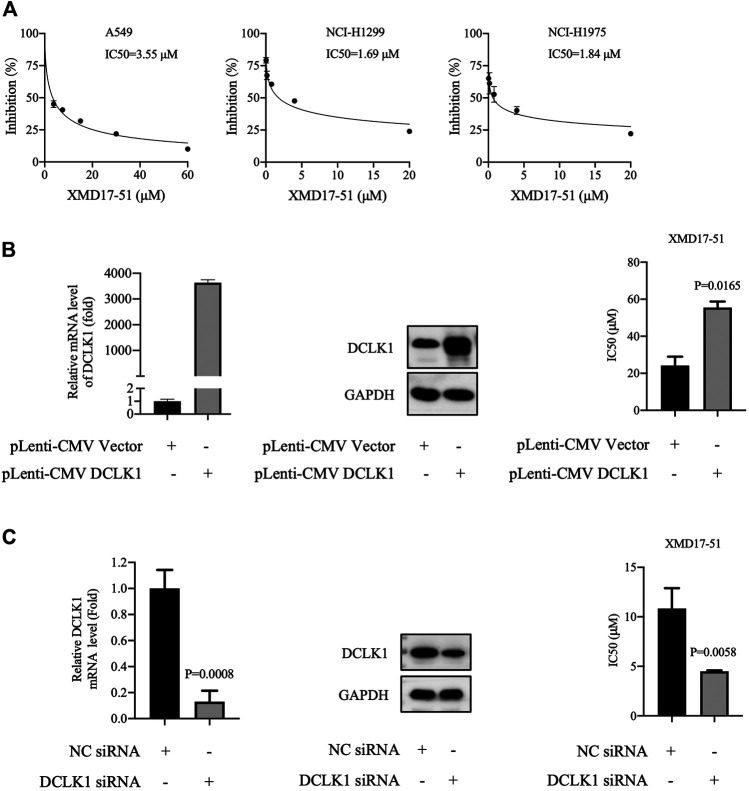
XMD-17-51 inhibits NSCLC cell proliferation via DCLK1. **(A)** XMD-17-51 inhibited A549, NCI-H1299 and NCI-H1975 cell proliferation, as detected by MTT proliferation assay. **(B)** DCLK1 overexpression impaired the anti-proliferative activity of XMD-17-51 in A549 cells. **(C)** DCLK1-knockdown A549 cells demonstrated a significantly decreased resistance to XMD-17-51. Data shown are representative of (graph) or are presented as the mean ± SD (blot) of at least three independent experiments. NSCLC, non-small cell lung carcinoma; DCLK1, doublecortin-like kinase 1; MTT, 3-(4,5-dimethylthiazol-2-yl)-2,5-diphenyl tetrazolium bromide.

To further explore the *in vitro* function of XMD-17-51 in NSCLC progression, its effects on A549, NCI-H1299 and NCI-H1975 cell proliferation were evaluated by MTT assay. As shown in Figure 2A, XMD-17-51 could inhibit A549, NCI-H1299 and NCI-H1975 cell proliferation in a significant dose-dependent manner, with an IC_50_ of 3.551, 1.693, and 1.845 μM, respectively.

As XMD-17-51 can modulate multiple protein kinases, the role of DCLK1 kinase in mediating the function of XMD-17-51 should be further determined. Next, DCLK1 was overexpressed in A549 cells by transfecting DCLK1 cDNA cloned inside pLenti-EF1a-EGFP-P2A-Puro-CMV vector, and the impact on XMD-17-5-induced cell proliferation delay was examined. As shown in Figure 2B, DCLK1 was successfully overexpressed in A549 cells. Subsequently, overexpressing DCLK1 promoted A549 cell resistance to XMD-17-51 treatment. The IC_50_ value of cells overexpressing DCLK1 was higher than that in the parallel-controlled cells. These results demonstrated that DCLK1 kinase activity confers resistance to XMD-17-51 inhibition.

On the contrary, the downregulation of DCLK1, mediated by siRNA-transfection, renders A549 cells sensitive to the antitumor activity of XMD-17-51 (Figure 2C).

### XMD-17-51 Inhibits EMT

**FIGURE 3:**
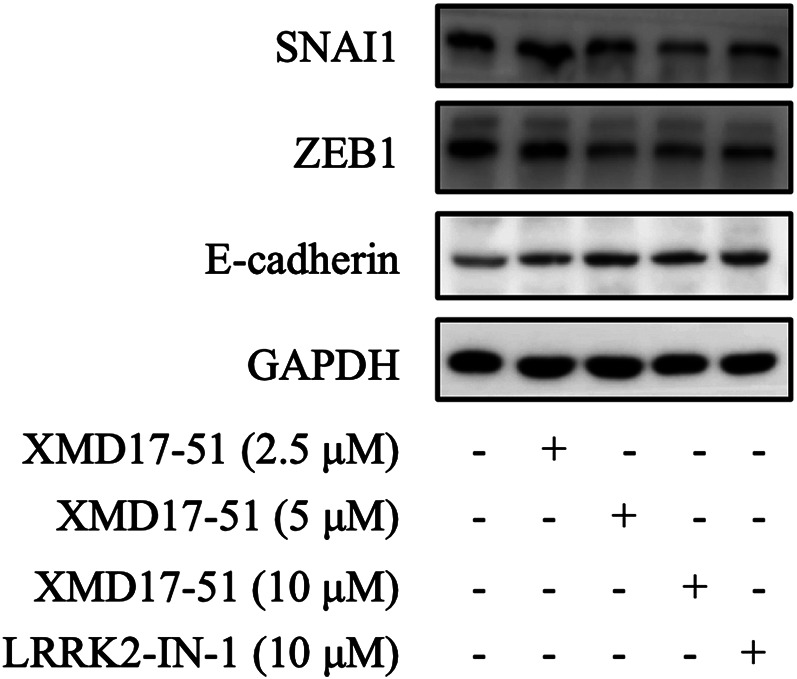
XMD-17-51 inhibits EMT in A549 cells. Snail1, ZEB1 and E-cadherin protein levels in A549 cells were detected using western blotting following treatment with XMD-17-51. GAPDH was used as a loading control. Data shown are representative of at least three independent experiments. EMT, epithelial-mesenchymal transition; ZEB1, zinc-finger-enhancer binding protein 1.

EMT is the key process driving cancer metastasis and DCLK1 is a regulator of EMT-related transcription factors. Therefore, the effects of XMD-17-51 on EMT were tested in A549 cells. As shown in Figure 3, XMD-17-51 decreased Snail-1 and ZEB1 protein levels, but increased those of E-cadherin. These results demonstrated that XMD-17-51 can significantly reduce the EMT process of A549 cells.

### XMD-17-51 Regulates Stemness of Lung Adenocarcinoma

**FIGURE 4:**
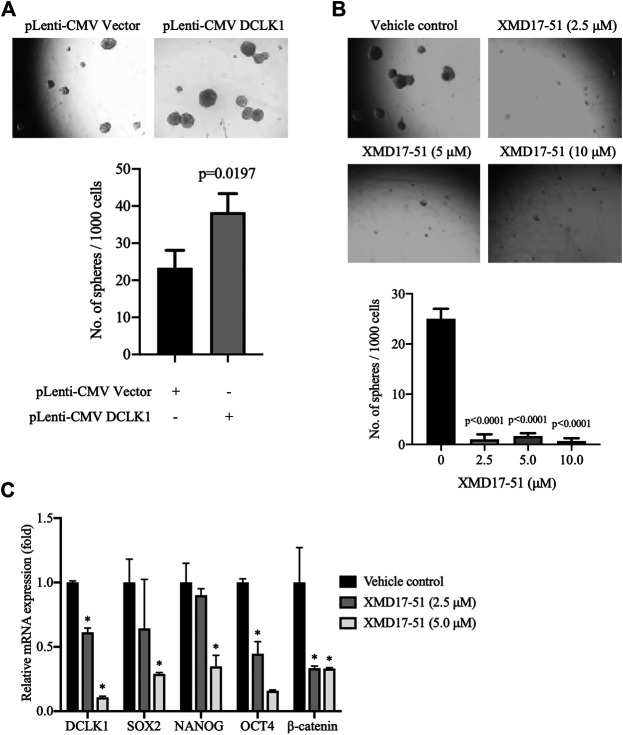
XMD-17-51 inhibits cancer cell stemness in A549 cells. **(A)** DCLK1 overexpression promoted sphere formation from A549 cells. Cells (1,000 cells/well) were suspended in stem cell medium. Spheres were counted 7 days after treatment and sphere formation efficiency is presented as the mean ± SD. **(B)** XMD-17-51 inhibited sphere formation from A549 cells. A549 cells (1,000 cells/well) were suspended in stem cell medium and treated with XMD-17-51 at different concentrations. Spheres were counted 10 days after treatment. Sphere formation efficiency is presented as the mean ± SD. **(C)** XMD-17-51 decreased cancer stem cell markers of A549 cells, as shown by RT-qPCR (normalization to GAPDH levels). Data are shown as a fold change relative to the levels in vehicle control cells and are presented as mean ± SD. DCLK1, doublecortin-like kinase 1.

**FIGURE 5:**
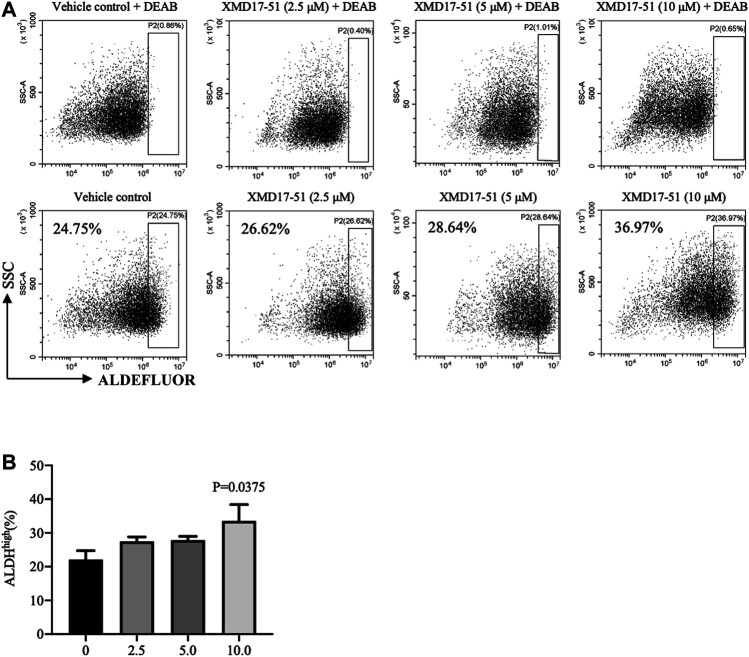
**(A)** As a functional readout of ALDH activity, A549 cells treated with different concentrations of XMD-17-51 were tested using the ALDEFLUOR assay. A specific inhibitor of ALDH, diethylaminobenzaldehyde or DEAB, was used as a negative control. **(B)** Higher XMD-17-51 concentration correlated with an increase in the number of ALDH^high^ cells. Data are presented as fold change relative to the levels in vehicle control cells and are presented as mean ± SD.

A well-known characteristic of CSC is their ability to survive and grow under non-adhesive conditions, the tumor sphere assay can be used to identity stem cells *in vitro* (O'Brien et al., 2009; Zeng et al., 2017). It was found by Ji et al. (2018) that, as compared with DCLK1^-^ cells, DCLK1^+^ cells can form compact tumor spheres, and possess an *in vitro* and *in vivo* self-renewal capability. Similarly, it was found herein that A549 cells overexpressing DCLK1 can form larger and more tumor spheres in the same number of days, as compared with cells transfected with empty vector (Figure 4A). To determine if XMD-17-51 can alter the tumor sphere-forming efficiency of lung cancer cells, A549 cells that were treated with different concentrations of XMD-17-51 were plated under low adherence conditions, and after ten days, tumor spheres were counted. The tumor sphere formation efficiency was significantly decreased in the drug treatment group in a dose-dependent manner, as compared with the control group (Figure 4B). To further explore the role of XMD-17-51 in the regulation of tumor stemness, the effect of different concentrations of XMD-17-51 was evaluated on the stem cell markers and pluripotency factors in NSCLC cells. XMD-17-51 reduced the expression of stemness markers such as β-catenin, and pluripotency factors such as SOX2, NANOG and OCT4. These results demonstrated that XMD-17-51 possesses anti-stemness properties (Figure 4C). However, the ALDH^+^ A549 cell percentage was increased by XMD-17-51 treatment in a dose-dependent manner (Figure 5). The exact underlying mechanisms need to be further studied. In the present study, we hypothesized that ALDH^+^ cells were more resistant to XMD17-51. As shown by the MTT experiment, XMD17-51 inhibited the proliferation of ALDH^−^ cells, resulting in an increase in the proportion of the remaining ALDH^+^ cells.

## Discussion

DCLK1 is highly expressed in several malignancies and is a marker of tumor stem cells that plays a critical role in the self-renewal capacity of cells derived from these tumors (Panneerselvam et al., 2020). Certain kinase inhibitors have been found to inhibit DCLK1 kinase activity, such as that of LRRK2-In-1, XMD-892 and DCLK1-IN-1; however, they are in the process of preclinical trials for cancer treatment. Herein, it was shown that XMD-17-51 could inhibit DCLK1 kinase, which may provide new insights into the design of small-molecule DCLK1 inhibitors. It was also shown herein that XMD-17-51 inhibits the proliferation of A549, NCI-H1299, and NCI-H1975 NSCLC cells. Due to XMD-17-51 being an inhibitor of multiple protein kinases, the antitumor activity of XMD-17-51 was tested in cells with DCLK1 knockdown or overexpression. DCLK1-overexpression was shown to impair the anti-proliferative activity of XMD-17-51 in A549 cell lines with an IC_50_ of 53.197 vs. 27.575 µM and vice versa. These results suggested that XMD-17-51 exhibited potent activity against NSCLC through the inhibition of DCLK1.

It was reported by Panneerselvam et al. (2020) that the knockdown of DCLK1 could prevent the ability of primary spheroids to form secondary spheroids. That result was consistent with the finding of the present study that XMD-17-51 not only inhibited DCLK1, but also reduced several stem cell proteins and pluripotency factors. XMD-17-51 significantly inhibited the sphere formation of A549 cells and downregulated NSCLC-related stem cell makers, such as β-catenin, NANOG and OCT4. However, the proportion of ALDH^high^ in A549 cells treated with XMD-17-51 was significantly increased and dose-dependent. The reason for this apparent difference may be associated with the fact that CSCs are plastic and express tissue-specific and cellular proliferation marker ALDH during MET, while they express CD44 and CD133 during EMT (Park et al., 2019; Han et al., 2020; Steinbichler et al., 2020). In addition, Charafe-Jauffret et al. (2009) reported that ALDH is a marker of epithelial proliferative breast CSC, but more mesenchymal stem cells are characterized by CD44^+^/CD24^−^ expression. CSC maintains the plasticity of transition between these states in the process of tumor microenvironment regulation. The present results demonstrated that XMD-17-51 inhibits EMT, which further supports the possibility of the drug changing the tumor microenvironment to increase ALDH^high^ in CSCs.

In conclusion, the present data indicated that XMD-17-51 inhibited DCLK1 kinase activity in a cell-free assay with an IC_50_ of 14.64 nM. In NSCLC cell lines, XMD-17-51 could also decrease DCLK1 protein levels and then inhibit cell proliferation, EMT and stemness. These results highlighted the potential use of XMD-17-51 as a DCLK1 inhibitor for the clinical treatment of lung cancer in the future.

## Data Availability

The raw data supporting the conclusions of this article will be made available by the authors, without undue reservation.
